# The role of Nrf2 in periodontal disease by regulating lipid peroxidation, inflammation and apoptosis

**DOI:** 10.3389/fendo.2022.963451

**Published:** 2022-11-22

**Authors:** Fengyu Ma, Shangdie Luo, Chunting Lu, Xinrong Jiang, Kexiao Chen, Jianwen Deng, Shuyuan Ma, Zejian Li

**Affiliations:** ^1^ Department of Stomatology, The First Affiliated Hospital of Jinan University, Guangzhou, Guangdong, China; ^2^ School of Stomatology, Jinan University, Guangzhou, Guangdong, China; ^3^ Department of Orthodontics, Huizhou Stomatological Hospital, Huizhou, Guangdong, China; ^4^ Science and Education Office, The First Affiliated Hospital, Jinan University, Guangzhou, Guangdong, China; ^5^ Chaoshan Hospital, The First Affiliated Hospital of Jinan University, Chaozhou, Guangdong, China

**Keywords:** Nrf2, periodontal disease, lipid peroxidation, apoptosis, bone homeostasis

## Abstract

Nuclear factor E2-related factor 2(Nrf2) is a transcription factor that mainly regulates oxidative stress in the body. It initiates the expression of several downstream antioxidants, anti-inflammatory proteins and detoxification enzymes through the Kelch-like ECH-associating protein 1 (Keap1) -nuclear factor E2-related factor 2(Nrf2) -antioxidant response element (ARE) signaling pathway. Its anti-apoptosis, anti-oxidative stress and anti-inflammatory effects have gradually become the focus of periodontal disease research in recent years. In this paper, the structure and function of Nrf2 pathway and its mechanism of action in the treatment of periodontitis in recent years were analyzed and summarized, so as to further clarify the relationship between Nrf2 pathway and oxidative stress in the occurrence and development of periodontitis, and to provide ideas for the development of new treatment drugs targeting Nrf2 pathway.

## 1 Introduction

Periodontal disease is among the most common chronic inflammations in the world, and plaque biofilm, as its initiating factor, affects the host’s immune function and inflammatory response. Inflammatory mediators produced by bacteria and their metabolites cause an imbalance of immune function and the destruction of periodontal tissue. A large number of studies have shown that periodontal disease is an oxidative stress disease (1), and defense cells such as polymorphonuclear neutrophils (PMN) will release reactive oxygen species (ROS) to eliminate pathogens when stimulated by bacteria and their metabolites, and excessive ROS will aggravate periodontal injury (2). *In vivo*, an anti-oxidation system is used to neutralize excessive active oxygen, and when the balance between oxidation and anti-oxidation *in vivo* is out of balance, oxidative stress will occur.

The transcription factor Nrf2 is a master regulator of genes encoding second-stage detoxification enzymes and anti-oxidative stress proteins that respond to electrophiles and oxidative stress (3). In the absence of this stimulation, Nrf2 is inactive because it is retained in the cytoplasm by Keap1 and rapidly degraded by the proteasomal system, rendering Nrf2 unable to bind to the nuclear antioxidant reaction element ARE, and mediates the transcription of various antioxidant enzymes and detoxification enzymes. Under the condition of oxidative stress, ROS causes the dissociation of Keap1 and Nrf2, thus releasing Nrf2 and transporting it to the nucleus (4). Then transcriptionally activate the expression of a series of antioxidant enzyme genes, such as heme oxygenase-1 (HO-1), superoxide dismutase (SOD), quinone oxidoreductase 1 (NQO1), glutathione peroxidase enzyme (GPX), glutamate-cysteine ligase (GCL), glutathione synthase (GSS), etc. (5). These regulated proteins can reduce the damage of lipid, DNA and protein caused by oxidative stress. However, besides redox homeostasis, Nrf2 also plays a key role in anti-inflammation, DNA repair, mitochondrial function, iron, lipid and glucose metabolism, cell proliferation, cell cycle and immune response (6, 7). Similarly, Nrf2 has a certain protective effect in periodontitis, mainly through anti-oxidative stress, inhibiting osteoclast activation, regulating periodontal cell proliferation, differentiation and apoptosis. In-depth exploration of the mechanism of Nrf2 in periodontal disease can provide better ideas and targets for research or clinical practice.

## 2 The regulation of Nrf2

### 2.1 Keap1-mediated regulation

**Figure 1:**
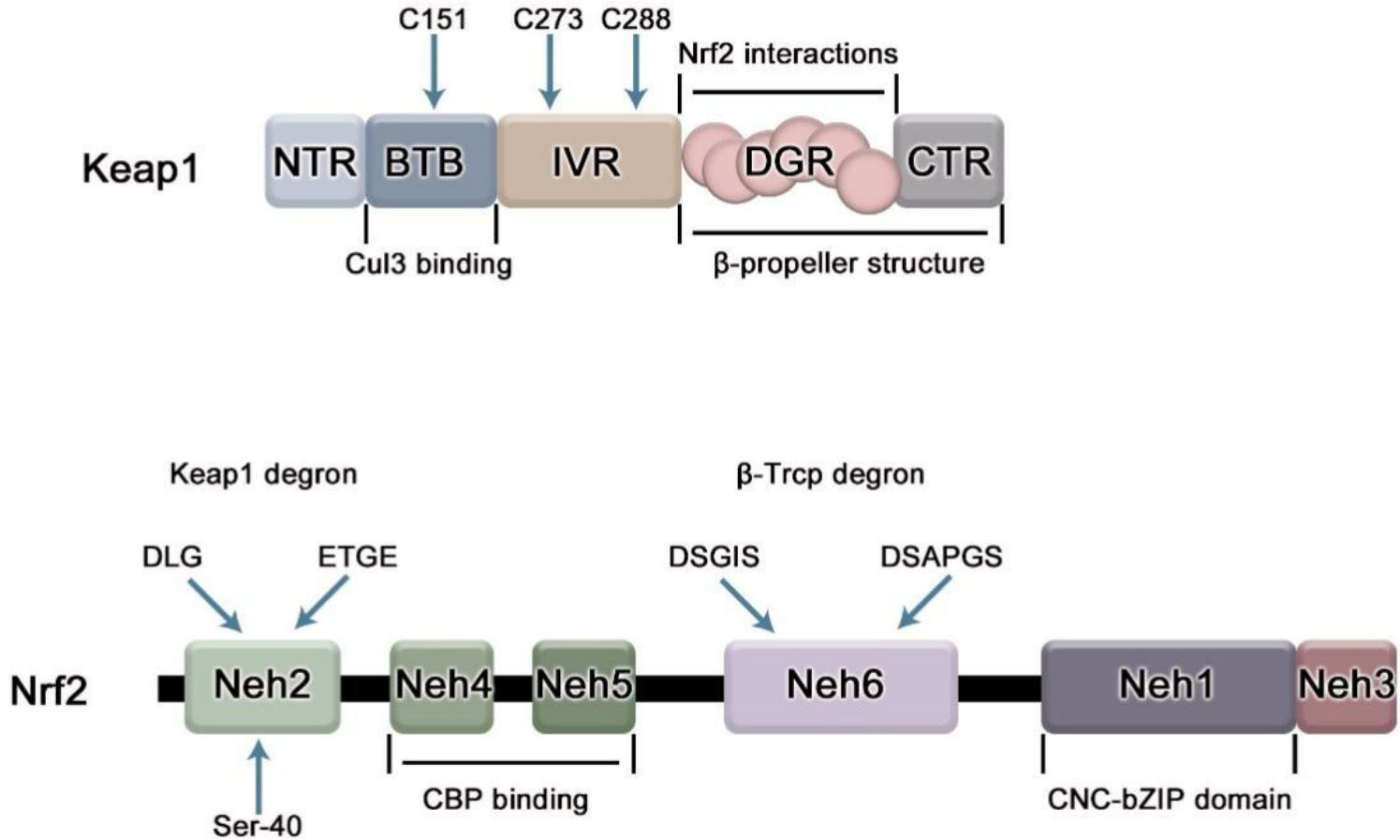
The domain structure of Nrf2 and Keap1. Keap1 has 5 domains. BTB domain mediates the homodimerization of Keap1 and its binding with Cullin3(Cul3), in which cysteine residue (C151) senses electrophilic compounds and mediates the dissociation of Nrf2 and Keap1; IVR region is related to the stability of Nrf2, and participates in the ubiquitination degradation of Nrf2 under non-oxidative stress. Cys 273 and Cys 288 on it are necessary to inhibit Nrf2; DGR domain mediates the binding of Keap1 to Nrf2. Nrf2 contains six domains, namely Neh1-6. The Neh1 domain contains a bZip motif, which mediates the dimerization of Nrf2 and sMaf, promotes the binding of Nrf2 and ARE, and is responsible for DNA recognition.; The Neh2 domain contains ETGE and DLG motifs required for interaction with Keap1; Neh3-5 acts as a trans-activation domain; The Neh6 domain contains DSGIS and DSAPGS motifs which can be recognized by β-TrCP linker protein.

Nrf2 is a member of the cap ‘n ‘collar(CNC) subfamily of alkaline leucine zipper transcription factors, which contains a highly conservative basic region-leucine zipper (bZIP) structure (**Figure 1**). Based on the conservative sequence of Nrf2, Nrf2 can be divided into six structural-functional domains, named Neh1-Neh6 (8). The Neh1 domain contains a CNC/basic leucine zipper domain that mediates the dimerization of Nrf2 with small Maf protein (sMaf), promoting Nrf2 binding to the ARE. The Neh2 domain, located at the N-most end of the gene sequence, contains two important conserved regions, DLG and ETGE. It is the region where Nrf2 combines with Keap1. Both motifs interact with the same residues of the Keap1 dimer (DGR/Kelch), but with different binding affinities, possibly due to the different composition of acidic residues. The affinity of the ETGE motif is 100 times that of DLG (3). The Neh3 domain is located at the C-terminal of Nrf2 and assists Nrf2 trans-activation, which is closely related to transcriptional activity (9). Neh4 and Neh5 are domains involved in the initiation of downstream gene transcription. The Neh6 domain contains the DSGIS and DSAPGS gene sequences and provides a binding site for β-TrCP, which mediates the Keap1-independent Nrf2 degradation pathway (10).

Kelch-like ECH-related protein-1 (Keap1) is a specific receptor for Nrf2 and the first major inhibitor of Nrf2, mainly present in the cytoplasm. Keap1 consists of five domains, namely NTR, BTB, IVR, DGR and CTR regions (**Figure 1**), among which NTR is located at the N-terminus of Keap1 and CTR is located at the C-terminus of Keap1, which are two amino acid terminals respectively. BTB, IVR, DGR are the main functional areas (11). The BTB region is a protein-protein interaction domain that mediates the homodimerization of Keap1, usually dimerizes with other BTB regions of Keap1, and binds to Nrf2 in a 1:1 ratio (12), the third cysteine residue C151 of the BTB domain is required for Nrf2 escape from Keap1-mediated repression (13). The IVR region is an oxidative stress-sensitive region, rich in cysteine, which is related to the stability of Nrf2 and is involved in the ubiquitination and degradation of Nrf2 under non-oxidative stress, in which two key cysteine residues C273 and C288 are required to inhibit Nrf2 nuclear aggregation (14); Keap1 binds to the N- terminal region of CUL3 through IVR domain and cooperates with Cul3-Roc1 complex to promote ubiquitination of Nrf2. The DGR domain (also known as the Kelch domain because it has 6 Kelch repeats), can recognize the two gene sequences ETGE and DLG of Nrf2, and negatively regulate the activity of Nrf2. DGR and CTR together constitute a β-helix structure, which is jointly defined as the DC domain that mediates the interaction with Nrf2Neh2 (15).

**Figure 2:**
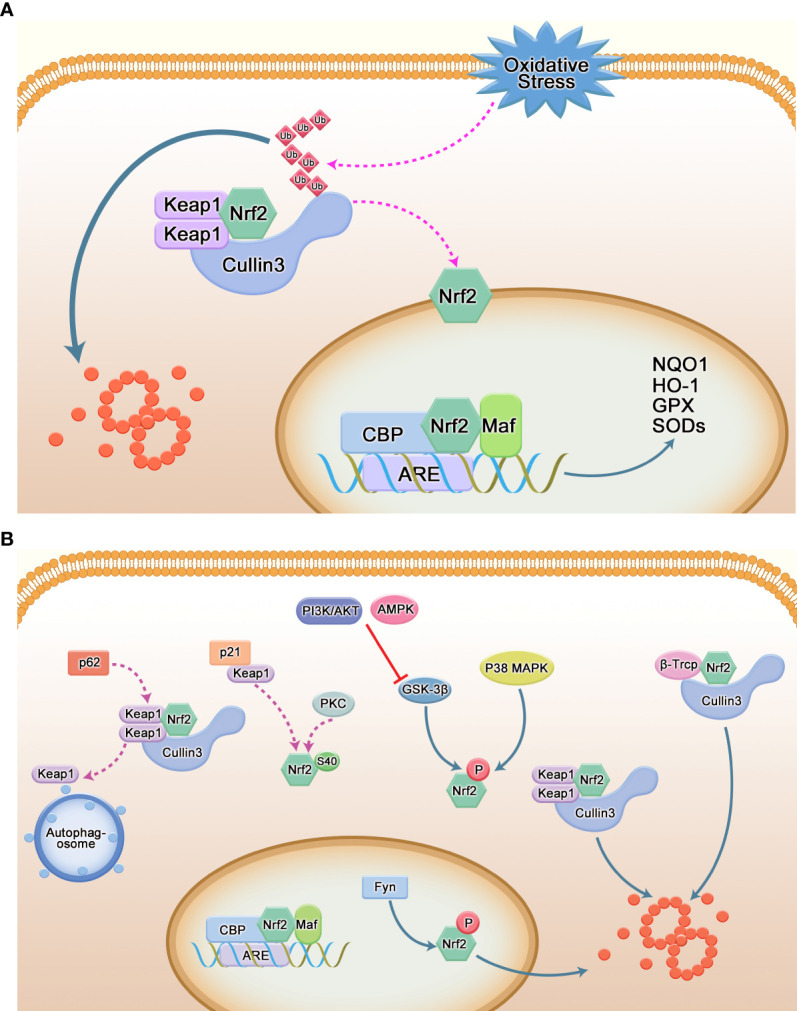
Regulation of Nrf2. **(A)** Canonical pathways regulated by Nrf2. Keap1 binds to Nrf2 in the form of dimers and mediates its ubiquitination degradation to maintain low levels of Nrf2 in the cytoplasm; under stress conditions, Nrf2 can dissociate from Keap1 and translocate to the nucleus to interact with AREs. After binding, the transcription of antioxidant enzymes is promoted. **(B)** Non-Keap1-mediated pathways. P62/SQSTM1 is an important selective autophagy protein. After binding to Keap1, it guides it into the autophagosome, leading to the autophagic degradation of Keap1; P21 interferes with the Keap1-Nrf2 interaction by binding to Keap1 and contributes to the positive regulation of Nrf2; p38 MAPK and GSK-3β can phosphorylate Nrf2 at Ser215, Ser408 and Ser577 and Ser334-338 of Neh6 domain, respectively, to facilitate its degradation; β-TrCP recognizes DSGIS and DSAPGS motifs to bind to Nrf2 and mediates its ubiquitination.

As shown in **Figure 2**, in the Keap1/Nrf2/ARE pathway, the inhibitory effect of Keap1 on Nrf2 is achieved by mediating its ubiquitination degradation. Ubiquitination is a hierarchical cascade of enzymatic reactions catalyzed by three enzymes – ubiquitin-activating (E1), ubiquitin-conjugating (E2), and ubiquitin-ligating (E3) enzymes (16), which is the major intracellular protein-degradation pathway. Under normal physiological conditions, the DGR region of Keap1 binds to the ETGE and DLG sequences of the Neh2 domain of Nrf2 and is anchored to the cytoplasmic actin cytoskeleton in the form of a Keap1-Nrf2 complex (17), which stabilizes Nrf2 in the cytoplasm. Keap1 containing BTB domain specifically binds to Cul3 to form E3 ligase complex, which is targeted to 26S proteasome for degradation, thus maintaining the low expression and non-activation of Nrf2 in cells. However, under pressure, oxidative stress and other conditions, oxidants or electrophilic compounds interact with C151 cysteine residues in the BTB domain of Keap1 (18), resulting in structural changes that separate it from Nrf2. After being activated, Nrf2 translocates into the nucleus and binds to ARE(also known as the electrophilic response element, is a cis-acting element that encodes many detoxification enzymes and cytoprotective protein genes in the promoter region) in the form of Nrf2-Maf. Neh4 and Neh5 bind to the co-activator CREB binding protein CBP, which will promote CBP to participate in the transcription activation of Nrf2 target gene, and initiate the expression of a series of downstream protective genes (19), such as various antioxidant enzymes and phase II detoxification enzymes, which enhance the detoxification and antioxidant capacity of cells, thus playing a protective role.

### 2.2 Non-Keap1-mediated regulation

In addition to the Keap1 signaling pathway, other signaling pathways can also affect the transcription of Nrf2 (**Figure 2**). For example, the second major repressor of Nrf2 that has been discovered is the β-transducin repeat-containing protein (β-TrCP) (11). The N-terminus and C-terminus of the Neh6 region contain the sequences DSGIS and DSAPG, respectively, which are similar to the common binding sequence of β-TrCP. Both the DSGIS motif and the DSAPGS motif were found to recruit β-TrCP and support the ubiquitination of Nrf2 by SCFβ-TrCP. In the cytoplasm, GSK-3β can also directly phosphorylate Ser335 and Ser338 of Nrf2 located in the Neh6 domain. This phosphorylated Nrf2 translocates to the nucleus and is directly recognized by the E3 ubiquitin ligase β-TrCP, triggering nuclear ubiquitination and degradation of Nrf2 (20). The PI3K/AKT pathway and AMPK pathway can negatively regulate or inactivate GSK-3β, and then indirectly regulate Nrf2, mediating its nuclear entry. In addition, protein kinase C (PKC) phosphorylates Ser40 in the Neh2 region of Nrf2, which induces dissociation of Keap1 from Nrf2, resulting in increased nuclear translocation of Nrf2. In addition, GSK-3β phosphorylates Src kinase Fyn at threonine residues in the nucleus leading to its nuclear accumulation, which in turn facilitates Nrf2 nuclear export (11). P38 MAPK can phosphorylate Ser215, Ser488, Ser577 in Nrf2, reducing its nuclear accumulation (21). p62 binds to Keap1 and interacts with the DLG and ETGE motifs of Nrf2 (22, 23); p21 interferes with Keap1-Nrf2 interaction by competing with Keap1 for the DLG motif of Nrf2 (24) and promotes Nrf2 pathway activation (25).

## 3 Nrf2 is involved in the regulation of periodontal disease

### 3.1 Nrf2, lipid peroxidation and periodontal disease

Among the factors that cause oxidative stress in cells, lipid oxidative modification in the lipid bilayer, especially lipid peroxidation, has become an important regulatory factor that determines the fate of cells (26). The cell membrane contains a large amount of polyunsaturated fatty acids esterified on phospholipids and free cholesterol. These lipids are the main targets of free radical attack. Lipid peroxidation (LPO) is lipid oxidative deterioration caused by ROS. LPO is a chain reaction, which mainly affects polyunsaturated fatty acids because there is a methylene bridge (-CH2-) with active hydrogen atoms. The chain reaction consists of three main steps: initiation, propagation and termination (27). Firstly, LPO needs to extract a hydrogen atom (28) from the diallyl position of polyunsaturated fatty acids in the lipid bilayer, and then form a phospholipid group (PL) with carbon as the center, then react with oxygen to generate phospholipid hydrogen peroxide radical (PLOO^•^), and remove a hydrogen from another PUFA to form PLOOH (29). Eventually, many products will be produced, including decomposition products of lipid peroxides such as 4-hydroxynonenal (4-HNE) and malondialdehyde (MDA) and oxidized and modified protein (30). This chain reaction ultimately leads to damage to the biofilm and altered fluidity (31). Overproduction of ROS and relative deficiency of antioxidants lead to increased oxidative damage to proteins in periodontal tissue and lipid peroxidation in plasma, saliva, and gingival crevicular fluid, these changes correlate with disease severity (2). Compared with healthy patients, the concentrations of TOS in serum, saliva and GCF in the chronic periodontitis group were significantly higher, and the clinical parameters were positively correlated with MDA and TOS levels in saliva and GCF. After treatment, the concentration of TOS in serum, saliva and GCF decreased (32). Similar findings were found in patients with periodontal disease with diabetes (33). Higher MDA levels were also found in human periodontal fibroblasts treated with butyrate, a metabolite of periodontal pathogens, compared with controls (34).

**Figure 3:**
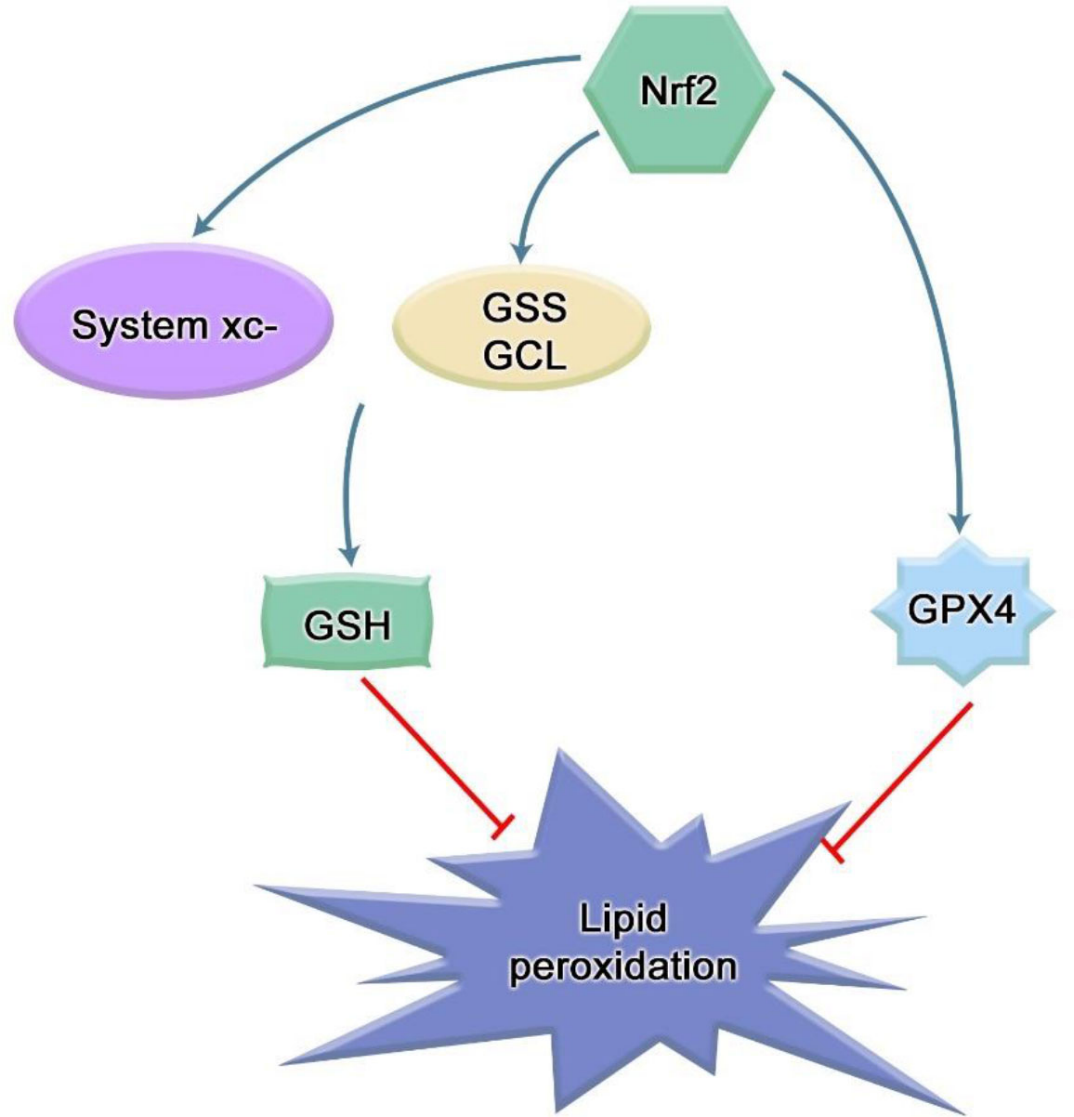
Mechanism of Nrf2 inhibiting lipid peroxidation. The cystine/glutamic acid reverse transporter on the cell membrane, namely, system xc-, is responsible for the uptake of cystine and the excretion of glutamic acid. The ingested cystine provides the raw material for intracellular GSH synthesis. Solute carrier family 7 member 11 (SLC7A11) is a part of this system, and SLC7A11 is a transcription target of Nrf2. As a cofactor of GPX4, GSH can cooperate with GPX4 to exert anti-lipid peroxidation effect. Many integrases involved in glutathione synthesis and metabolism are under the control of Nrf2, such as glutamate-cysteine ligase (GCL) and glutathione synthase (GSS). In addition, Nrf2 can stimulate the expression of GPX4.

Cells also have a system to prevent LPO. Glutathione (GSH)/glutathione peroxidase 4(GPX4) can eliminate lipid peroxide (35), effectively counteract lipid bilayer peroxidation and prevent cell membrane damage. The cystine/glutamate reverse transporter on the cell membrane, the system xc-, mediates the generation of glutathione. GPX4 uses GSH as a reducing agent, which can significantly reduce the accumulation of LPO in cells. In contrast, Nrf2 can directly control glutathione synthase (GSS) and glutamate-cysteine ligase (GCL) to tightly regulate GSH levels (36). Cysteine is produced by the reduction of cystine, which is imported into cells through the system xc- (11, 22). Nrf2 increases cysteine supply by directly activating Slc7a11, the gene encoding the xCT subunit of the system xc-. In addition to GSH synthesis, Nrf2 also plays a role in GSH maintenance. Nrf2 regulates the transcription of many ROS detoxification enzymes, such as glutathione peroxidase and several glutathione S- transferase, which use GSH to inactivate ROS (**Figure 3**). In a randomized clinical study, Tsai et al. observed that the concentration of GSH in the saliva of healthy patients was significantly higher than that of periodontitis patients, and the concentration of GSH increased after routine periodontal therapy, called scaling and root planning (SRP). This suggests that higher GSH concentrations are associated with better periodontal condition (37). Similarly, several studies have observed significantly higher levels of MDA detected in saliva in patients with periodontitis who have not received periodontal treatment (38), and anti-photodynamic therapy can increase the content of GSH in saliva while decreasing the content of MDA (39). Furthermore, human periodontal ligament fibroblasts treated with butyrate also showed a decrease of GSH and GPX4 (34). Lipid peroxides were also elevated in experimental mouse periodontitis models, and decreased Nrf2 expression was closely related to the aggravation of periodontal damage and oxidative damage in diabetic periodontitis (40). In the oxidative stress state of periodontitis stimulated by hydrogen peroxide treatment of human periodontal ligament stem cells (PDLSCs), it was found that hydrogen peroxide stimulation increased the content of MDA in PDLSCs, and the mRNA and protein levels of Nrf2 were up-regulated with an increase in its downstream effectors, heme oxygenase-1 (HO-1), NAD(P)H: quinone oxidoreductase 1 (NQO1) and γ-glutamyl cysteine synthetase (γ-GCS) as the exposure concentration increases (41). Moreover, Carlson et al. found that many bacteria in the oral cavity and periodontal pocket can also consume GSH (42). These pieces of evidence indicate that lipid peroxidation exists in periodontitis, and Nrf2 may play an important role in it.

### 3.2 Nrf2 regulates periodontal cell apoptosis

**Figure 4:**
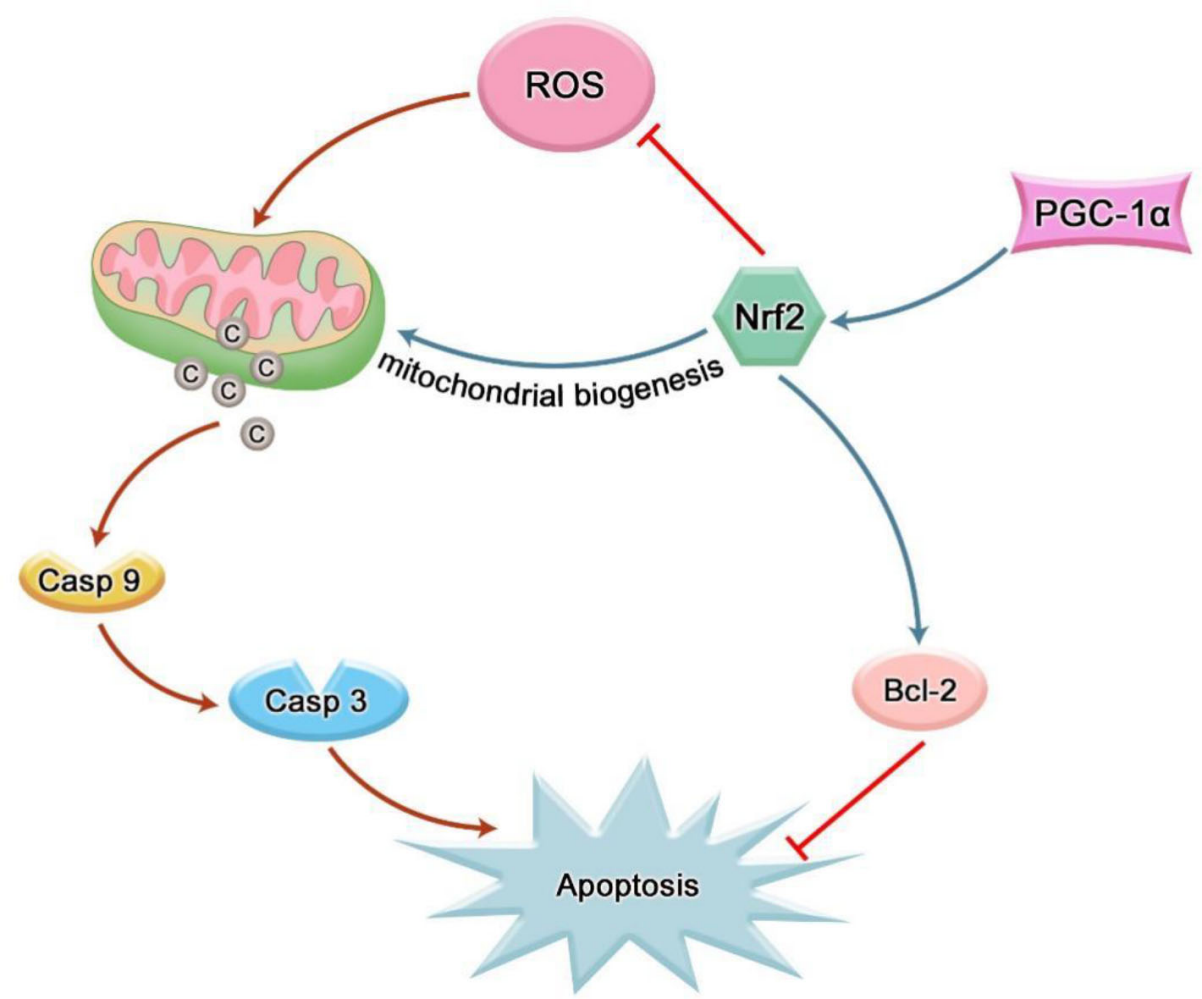
Nrf2 can inhibit cell apoptosis by reducing intracellular ROS; Nrf2 can indirectly inhibit cell apoptosis by participating in mitochondrial biogenesis; Nrf2 can also inhibit apoptosis by directly mediating the transcription of anti-apoptotic factors.

Recent studies suggest that the direct and indirect involvement of oxidative stress is the key factor leading to the destruction of periodontal tissue. Physiological levels of ROS can act as second messengers to regulate physiological activities such as intracellular homeostasis and signal transduction, while excess ROS exerts cytotoxicity and induce apoptosis in tissue cells (43). Many studies have shown that ROS can participate in the occurrence and development of periodontitis by inducing apoptosis of periodontal tissue cells. It is found that nicotine can induce human gingival fibroblasts (HGFs) to produce excessive ROS, and a higher level of phosphorylated JNK can be detected in a short time. Subsequently, the structure and function of mitochondria changed to release cytochrome c (cyt c), which activated the cascade reaction of caspase-3 and caspase-9 to induce HGFs apoptosis (44). ROS can also directly attack DNA and cause apoptosis. 8-hydroxy-deoxyguanosine (8-OHdG) is the most common stable product of ROS-mediated DNA damage (45). A study reported that the levels of 8-OHdG in whole saliva and GCF of patients with chronic periodontitis (CP) who had teeth with poor prognosis in the Japanese population were significantly higher than those in the control group (46). Takane et al. found that the level of 8-OHdG in the whole saliva of patients with periodontitis was significantly higher than that of healthy subjects, while the level of 8-OHdG in the gingival blood of patients with aggressive periodontitis was higher than that of CP patients, suggesting ROS damage correlated with disease severity (47). Mitochondria are the main source of ROS and the main target of ROS, and mitochondrial DNA is most vulnerable to ROS attack (48). Sugano et al. collected gingival tissue samples from patients with chronic adult periodontitis during flap surgery and analyzed them using polymerase chain reaction technology. They found mitochondrial DNA deletions in the gingival tissue of these patients, but not in healthy patients (49). Another study found that 5-kbp mtDNA deletion was detected in gums of CP patients, but not in healthy controls. Furthermore, the occurrence of 5-kbp mtDNA deletion was significantly correlated with all clinical parameters (plaque index, gingival index, clinical adhesion level and probing depth) (50). Nrf2 could increase the expression of antioxidants in MSCs treated with hydrogen peroxide and reduce apoptosis (51). The anti-apoptosis effect of Nrf2 on PDLSCs under oxidative stress was found by silencing and over-expressing PDLSCs with Nrf2 (41). In addition, the induction of apoptosis of scalp cortical neurons by cypermethrin was also proved to be reversed by Nrf2/ARE pathway (52). CUL3 can promote inflammation and apoptosis of PDLSCs by down-regulating the expression of Nrf2 (53). Mitochondria are indispensable organelles in the apoptosis mechanism. Activation of Nrf2 leads to mitochondrial biogenesis (**Figure 4**) (54). Mitochondrial biogenesis is the process by which cells increase the mass and copy number of individual mitochondria and is regulated by peroxisome proliferator-activated receptor-γ coactivator 1α (PGC-1α)-nuclear respiratory factor-1 (Nrf1)-nuclear respiratory factor 2 (Nrf2) Regulation of the mitochondrial transcription factor A (TFAM) pathway (55). Y.k. Chang et al. have observed that the amount of mitochondrial DNA in Nrf2-deficient livers is significantly lower than that in wild-type cells (56). Significantly enhanced mitochondrial ROS production was detected in periodontal tissues of diabetic rats with periodontitis. The mRNA expression levels of PGC-1α, Nrf2 and TFAM in the periodontitis group decreased and showed a higher level of apoptosis (57), which clearly proved that the aggravation of mitochondrial OS might interfere with the enhancement of periodontal cell apoptosis in diabetic periodontitis. Moreover, studies have shown that Nrf2 can not only combine with an ARE between nucleotide positions −608 to −600 to induce the expression of the Bcl-xL gene, but also bound to ARER3 in thepromoter region of the Bcl-2 gene after Nrf2 is transferred to the nucleus, increasing the Bcl-2 gene transcription, thus realizing the direct regulation of apoptosis by Nrf2 (**Figure 4**) (58, 59).

### 3.3 The role of Nrf2 in inflammatory response of periodontal disease

The pathogenesis of periodontal disease is largely the result of the host response to microbial-induced tissue destruction. Dysregulation of local microbes interacting with host cells leads to hyperactivation of the host immune response. Therefore, it is the host response that leads to tissue destruction. Host cells include periodontal cells and other immune cells that exert pro- or anti-inflammatory effects. The direct infiltration of many mononuclear immune cells such as monocytes, macrophages, lymphocytes, and plasma cells, as well as the production of inflammatory cytokines, lead to chronic inflammation. Understanding the regulatory role of Nrf2 in these immune cells and expression of cytokines by these cells has the potential to help develop new treatments for periodontitis.

#### 3.3.1 Nrf2 and inflammatory mediators

**Figure 5:**
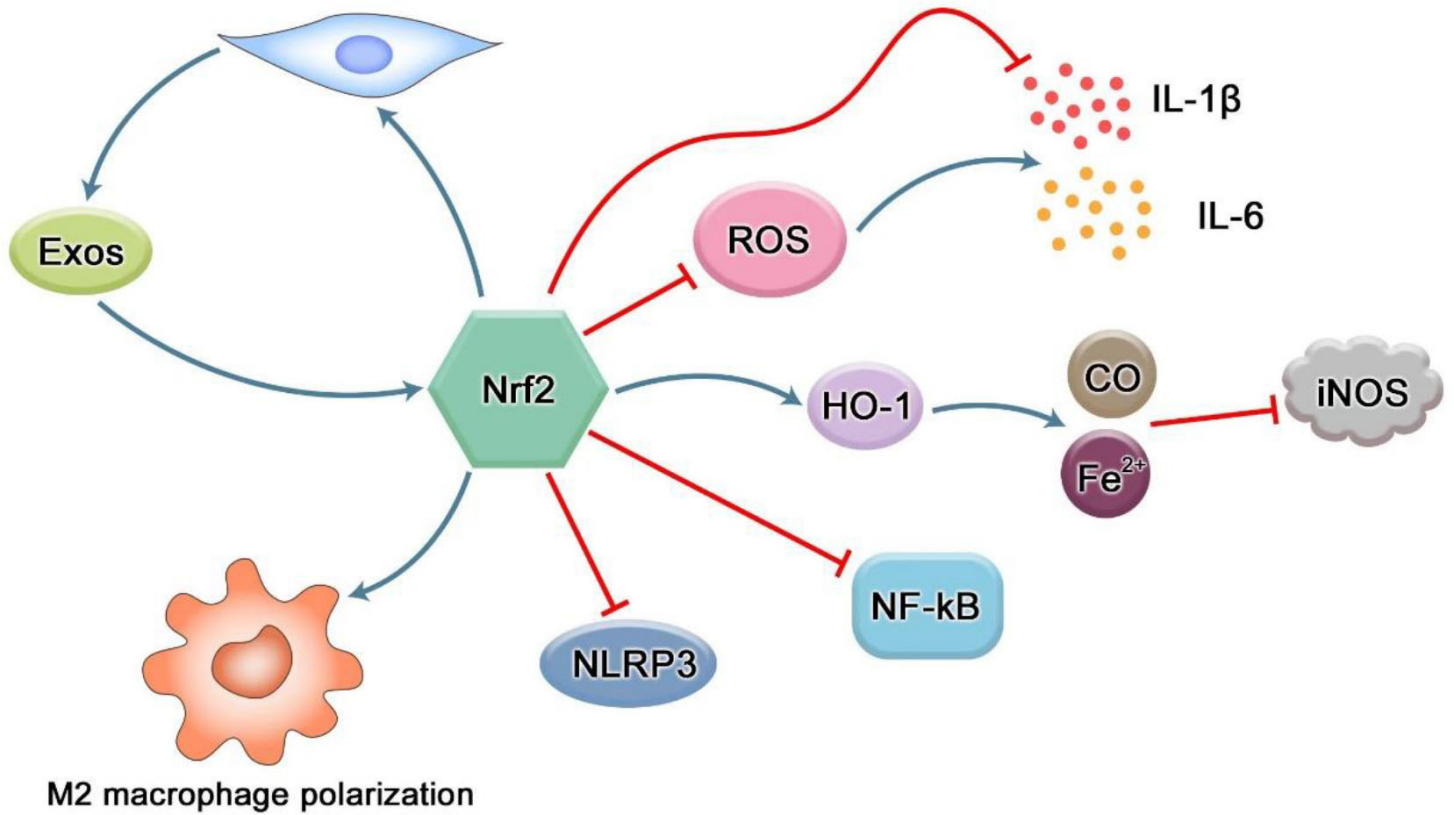
Regulation of Nrf2 in inflammation. The production of ROS can induce high expression of pro-inflammatory cytokines such as IL-1β and IL-6. However, Nrf2 not only reduces the production of pro-inflammatory cytokines through its antioxidant effect, but also directly inhibits the expression of inflammation-related genes. iNOS can be induced to express in various inflammatory diseases and play the role of inflammatory mediators. The downstream factor of Nrf2, HO-1, can negatively regulate iNOS through CO-mediated iNOS inactivation and ferrous iron inhibiting iNOS transcription. NF-κB is an important transcription factor involved in regulating the expression of inflammation-related genes in the body. There is a crosstalk between the Nrf2 pathway and NF-κB, and Nrf2 can inhibit its activation. As the most typical inflammasome in pyroptosis, NLRP3 can promote the maturation and secretion of a variety of inflammatory mediators, and produce an inflammatory response. However, Nrf2 pathway can reduce the inflammatory response by inhibiting the activation of NLRP3 inflammasome. Nrf2 can reverse the transformation of pro-inflammatory M1 macrophages into anti-inflammatory M2 macrophages. Nrf2 can enhance the proliferation and differentiation potential of mesenchymal stem cells, thereby increasing the number of exosomes and promoting tissue repair. In turn, exosomes can promote the expression of Nrf2 and exert antioxidant effects to reduce inflammation and oxidative stress.

Periodontitis is a chronic infectious disease of periodontal supporting tissues caused by plaque microorganisms, which can lead to inflammation and destruction of periodontal supporting tissues. The anti-inflammatory effect of Nrf2 is firstly exerted through its antioxidant effect (60). ROS is a key signal molecule that plays an important role in the progression of inflammatory diseases. The enhancement of ROS produced by polymorphonuclear neutrophils (PMN) in inflammatory sites will lead to endothelial dysfunction and tissue damage (61). Studies by Cavalla et al. showed that ROS can increase the activity of gelatin-soluble matrix metalloproteinases (MMPs) and stimulate the secretion of pro-inflammatory cytokines (IL-1β, IL-6, etc.) from human periodontal ligament fibroblasts (HPDLFs), thereby aggravating the destruction of periodontal tissue (62). The analysis of polymorphonuclear neutrophils (oPMN) in the oral cavity showed that compared with the periodontal healthy control group, the expression of Nrf2 in patients with severe chronic periodontitis was significantly reduced, and the Nrf2 knockout mice showed severe local oxidative damage and increased alveolar bone and attachment loss at the site of periodontitis (63). Schisandra chinensis α-isocarbophenol induced HO-1 expression through Nrf2 signal, and enhanced the anti-inflammatory activity of macrophages stimulated by Porphyromonas gingivalis lipopolysaccharide (64). Resveratrol enhanced the induction of HO-1 through Nrf2 signal, and then inhibited the expression of pro-inflammatory factors in gingival fibroblasts stimulated by LPS (65). Isorhamnetin upregulates the expression of Nrf2 and HO-1 in gingival fibroblasts, and the anti-inflammatory effect of isorhamnetin is blocked when Nrf2 is knocked out (66). These results suggest that Nrf2 and its downstream products may be involved in the pathological process of periodontitis through its antioxidant effect. Nitric oxide synthase iNOS increases in periodontitis, and NO produced by iNOS participates in the progress of periodontitis (67, 68). NO easily reacts with superoxide to become peroxynitrite, which is a powerful RNS that further damages tissues and represents the combination of ROS and NO pathways (69). HO-1 catalyzes heme to carbon monoxide (CO), free ferrous iron and biliverdin. The expression of HO-1 can negatively regulate iNOS through CO-mediated inactivation of iNOS and inhibition of iNOS transcription by ferrous iron (**Figure 5**) (70). In addition, some data show that there is a crosstalk between Nrf2 and NF-κB pathways. Nrf2-dependent HO-1 expression can inhibit the secretion of NF-κB by human umbilical vein endothelial cells stimulated by TNF-α (71). In the male SD rat liver transplantation model, the up-regulation of HO-1 expression mediated by activated Nrf2 leads to the inhibition of NF-κB signal (72). NF-κB is a key transcription factor in the pathogenesis of inflammation (73). Under non-stimulatory conditions, NF-κB binds to IkB-α inhibitors as a homodimer or isodimer complex of p50 and P65 subunits in cytoplasm under unstimulated conditions (74). Once activated, IkB-α is rapidly degraded by IκB kinase β (IKKβ) -mediated phosphorylation, leading to dissociation of the complex (75). The released NF-κB then translocates into the nucleus, where it activates the transcription of several inflammatory genes. At present, it is considered that the influence of NF-kB activity on Keapl/Nrf2/ARE signaling pathway mainly lies in three aspects.: firstly, Keap1 can also mediate IKKβ ubiquitination through E3 ubiquitin ligase, thus inhibiting NF-ĸB activity (76). Secondly, the Nrf2 pathway increases HO-1 expression and antioxidant defense to inhibit NF-ĸB activation, thus neutralizing ROS and detoxification chemicals. As a result, ROS-related NF-ĸB activation was inhibited. Thirdly, NF-ĸB reduces free CBP through competitive binding with CH1-KIX domain of CBP, and promotes phosphorylation of p65 at the Ser276 site. Because CBP preferentially interacts with phosphorylated p65 at Ser276, the overexpression of p65 limits the availability of CBP in Nrf2 interaction (77, 78). Activation of the NF-ĸB pathway also exists in periodontal disease. In the mouse RAW264.7 macrophage model stimulated by Porphyromonas gingivalis lipopolysaccharide, magnolol inactivates NF-ĸB by activating the Nrf2/HO-1 pathway (79).

#### 3.3.2 Nrf2 and pyroptosis

Pyroptosis is a newly discovered mechanism of programmed cell death of inflammatory cells mediated by caspase-1, which is mainly characterized by cell lysis and the release of a large number of pro-inflammatory factors (80). There are classical and non-classical pathways for pyroptosis. The canonical pathway is mainly mediated by activation of caspase-1 by NLRP3 inflammatory bodies., while the non-canonical pathway is mainly mediated by caspase-4 and -5 in humans or caspase-11 in mice. Both pathways cleave gasdermin D (GSDMD), a member of the Gasdermin family, to release its gasdermin-N domain, which penetrates the plasma membrane to induce cell swelling and osmotic lysis. Finally, cytoplasmic molecules, such as interleukin -1β(IL-1β) and -18(IL-18), are released from the pores formed by GSDMD, causing a strong inflammatory reaction (81). The redox signal is an important mechanism that mediates the activation of NLRP3 inflammatory corpuscles. Xiuting Liu et al. found that rotenone (a respiratory chain inhibitor that can increase mitochondrial ROS and total ROS) induced higher inflammasome activity of NLRP3 compared with lipopolysaccharide, indicating that ROS promoted the initiation step of NLRP3 assembly, and Nrf2 activation-induced NQO1 expression to clear ROS, thus inhibiting NLRP3 expression and subsequent caspase-1 cleavage (**Figure 5**) (82). Idecalcitol, a vitamin D analog, reduces LPS-induced NLRP3 inflammasome-dependent pyroptosis in human gingival fibroblasts *via* the Nrf2/HO-1 pathway (83).

#### 3.3.3 Regulation of Nrf2 in macrophages

Macrophages are among the first immune cells to respond to bacteria and their products. It is found that Nrf2 binds to the vicinity of 203 genes (including IL-6 and IL-1β) in macrophages and inhibits the recruitment of RNA Pol II. The pathways related to the inflammatory diseases were enriched in these genes, supporting the notion that Nrf2 contributes to the resolution of inflammation (84). M1 macrophages have a significant pro-inflammatory effect, and participate in the inflammatory reaction and tissue damage of periodontal tissue (85). Nrf2 can reverse the polarization of M1-type macrophages and turn to the polarization of M2-type macrophages (**Figure 5**) (86). The polarization of macrophages to the M2 type can promote healing and eventually promote alveolar bone regeneration (87). Mesoporous Prussian Blue (MPB) nanoparticles loaded with BA (abbreviated as MPB-BA) could up-regulate Nrf2 to remove ROS, inhibit the nuclear factor κB(NF-κB) signaling pathway, and transform the macrophage phenotype from M1 to M2, thereby reducing the inflammatory response (88).

#### 3.3.4 Nrf2 and exosomes

Exosomes are extracellular vesicles containing a variety of molecular components secreted by cells. They are small lipid bilayer particles that are secreted by budding from the plasma membrane and released into the extracellular space. These vesicles carry proteins, mRNAs and miRNAs between adjacent cells, transmit a large amount of information and stimuli, and can perform the functions of intercellular material transport and signal communication through autocrine and paracrine effects (89). Almost all cells can produce exosomes, and the effect of exosomes is related to the cells from which they are derived. For example, exosomes from P. gingivalis can significantly stimulate macrophages to produce inflammatory mediators such as tumor necrosis factor-α, and induce the activation of macrophage inflammatory bodies (90). Actinobacter actinobacter (Aa) exosomes containing white toxin are highly toxic and can specifically kill host immune cells (91). M2-type macrophage-derived exosomes can enhance the osteogenic differentiation of mesenchymal stem cells, while M1-type macrophage-derived exosomes inhibit the osteogenic differentiation of MSCs (92). Mesenchymal stem cells (MSCS) are ideal candidates for regenerative medicine. In addition to their multidirectional tissue-cell differentiation, MSCS also have paracrine function. A growing number of studies have shown that stem cell exosomes can regulate the expression of Nrf2 and HO-1 by targeting Keap1 (**Figure 5**). MSC-Exo has been reported to ameliorate oxidative stress-induced skin damage by attenuating cellular and tissue responses to inflammation and oxidation; Adipose-derived stem cell (ADSCs) exosome-treated macrophages upregulate HO-1 expression by inhibiting Keap1 and promoting Nrf2 expression and translocation into the nucleus, thereby reducing inflammation and oxidative stress (93). It is reported that the differentiation of MSCs is often accompanied by mitochondrial biogenesis, which is controlled by PGC-1 α and further activates Nrf1, Nrf2 and mtTFA expression in coordination with γ polymerase to promote mtDNA replication. The increased expression of Nrf1 and Nrf2 can increase the mitochondrial biogenesis of hBMSCs, thus enhancing the osteogenic differentiation potential of hBMSCs (94, 95). FNDC5-pretreated BMSC-Exos were also shown to have anti-inflammatory effects and promote M2 macrophage polarization through the NF-κB signaling pathway and the Nrf2/HO-1 axis (96). Inflamm-aging is closely related to the pathogenesis of periodontitis. The vicious circle of free radicals–inflammation–aging–free radicals during oxidative stress accelerates the aging process. It has been proposed that antioxidant capacity mediated by the Nrf2 pathway is the key to maintain redox metabolism during aging (97). Recent evidence shows that human placental mesenchymal stromal cell-derived exosomes can attenuate CD4+ T cell senescence by targeting Nrf2-mediated antioxidant defense (98).

#### 3.3.5 Regulation of bone homeostasis by Nrf2

**Figure 6:**
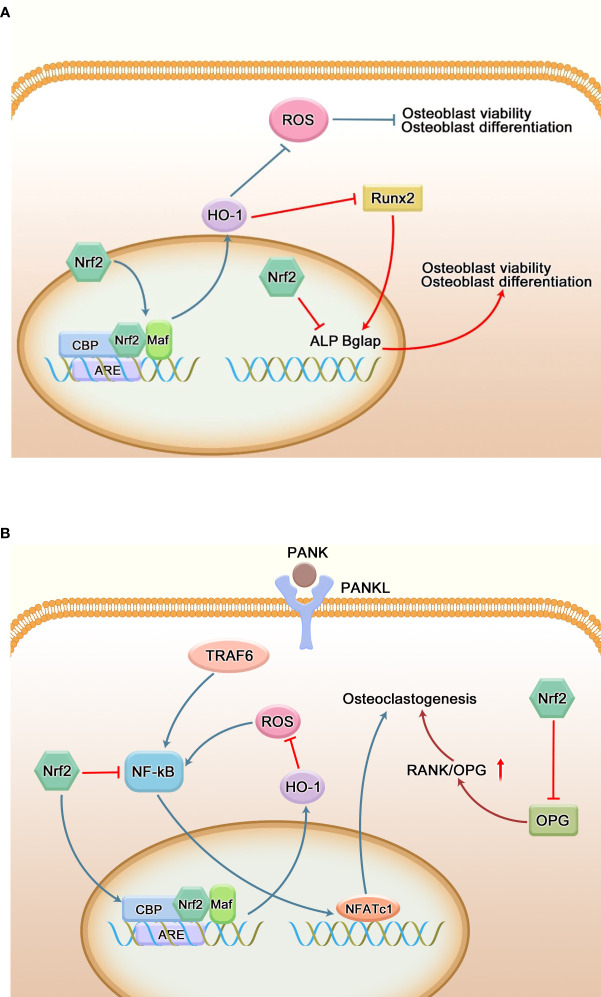
Regulation of bone homeostasis by Nrf2. **(A)** Regulation of osteoblasts by Nrf2. Oxidative stress hinders the proliferation and differentiation of osteoblasts, resulting in osteoblast dysfunction. Nrf2 is able to reduce peroxidation through its antioxidant capacity, thereby reducing osteoblast damage and maintaining its function. Runx2 is a transcription factor necessary for osteoblast differentiation, and overexpression of Nrf2 and its downstream factor (HO-1) interacts with Runx2; it can also directly inhibit the expression of many key genes regulating osteogenic differentiation and mineralization to negatively regulate osteogenic differentiation. **(B)** Regulation of osteoclasts by Nrf2. RANKL regulates the mature differentiation of osteoclasts by binding to RANK on the surface of osteoclast precursor cells. ROS can directly or indirectly promote the differentiation of osteoclast precursor cells into osteoclasts. The Nrf2/ARE signaling pathway ultimately inhibits the activity of the key osteoclast transcription factor NFATC1 by regulating the production of ROS and the expression level of NF-κB and other related signaling pathways, thereby inhibiting the differentiation of osteoclasts. Meanwhile, Nrf2 can also indirectly promote the formation of osteoclasts by inhibiting the secretion of OPG by osteoblasts and increasing the ratio of RANKL/OPG.

Alveolar bone resorption can occur in the advanced stage of periodontitis, leading to tooth loss. The balance between osteogenesis and osteoclast is the key to maintain the alveolar bone homeostasis. The direct factor for alveolar bone resorption in periodontitis patients is the increase of osteoclasts and their active bone resorption. More and more studies have found that oxidative stress is closely related to bone destruction, and ROS can induce osteoclastogenesis and differentiation (99, 100). As shown in **Figure 6**, there are two opposing voices in the study of the effects of Nrf2 on osteoclasts. Nrf2 not only inhibits osteoclast differentiation by regulating peroxidation and reducing reactive oxygen radicals, but also inhibits osteoclast differentiation by inhibiting NF-κB, c-Fos and NFATc1 (20). Sulforaphane (SFN) and epigallocatechin gallate (EGCG) enhance the nuclear translocation of Nrf2 and the expression of anti-oxidative stress kinases such as HO-1 to weaken intracellular ROS and remarkably inhibit RANKL-mediated osteoclastogenesis *in vitro*, thereby inhibiting the orthodontic tooth movement (101). Resveratrol can reduce the production of circulating ROS through the Nrf2 pathway, inhibit the formation of osteoclasts in rats, and ameliorate lipopolysaccharide-mediated alveolar bone loss in rats (65). Hiroyuki Kanzaki et al. found that Keap1/Nrf2 axis controlled intracellular ROS levels through transcriptional regulation of cytoprotective enzyme expression, and played a major role in regulating RANKL-mediated osteoclast formation. Local Nrf2 overexpression inhibited gene expression of these osteoclast markers and osteoclast factors (MMP9, TRAP, RANKL, TNF) induced by LPS, and bone resorption of mouse skulls (102). Paeonol down-regulates NF-kB activation and osteoclast production by enhancing Nrf2 expression (103). In contrast, Cheol Kyu Park et al. found that Nrf2 indirectly supports osteoclast formation by negatively regulating OPG induction by directly binding to its OPG promoter (104).

The role of Nrf2 in osteoblast differentiation and activity remains controversial. Some studies have found that Nrf2 has a positive regulatory effect on the differentiation of osteoblasts. Siqi Ying et al. found that low-intensity pulsed ultrasound could reduce the inhibition of hydrogen peroxide on the activity of human periodontal ligament cells and osteogenesis through the Nrf2 pathway (105). Panax Ginseng fruit can induce osteogenic differentiation in *in vitro* and *in vivo* periodontitis models by regulating Nrf2/HO-1 signaling pathway (106). Xiong et al. found that targeted interference with Nrf2 could effectively inhibit the expression of osteogenic related proteins and genes ALP, COL1, and Runx2, and further studies showed that curcumin could mediate the osteogenic differentiation of periodontal ligament cells by activating PI3K/AKT/Nrf2 signaling pathway (107). Nrf2 activator T-BHQ promoted the osteogenic differentiation of PDLSCs under cyclic mechanical stretching, and enhanced the expression level of osteogenic markers in orthodontic rats. Micro-ct was used to observe that T-BHQ improved the microstructure of alveolar bone during orthodontic tooth movement in rats (108, 109). Metformin can promote the osteogenic differentiation of PDLSCs by activating Akt/Nrf2 signaling pathway (110). *In vitro*, deferoxamine also promotes the osteogenic differentiation of periodontal cells by activating the Nrf2 pathway (111). On the contrary, studies have found that the overexpression of Nrf2 and its downstream factor (HO-1) can interact with Runx2, an important transcription factor during osteogenic differentiation (112), or directly bind to the ARE-like sequence near OSE2 in the osteocalcin promoter, thereby reducing the transcriptional activity of osteocalcin (113). H_2_O_2_ increases the induction of Nrf2 protein in DAG-exposed human periodontal ligament fibroblasts. However, WNT-1 treatment significantly inhibited this increase. Nrf2 silencing mediated by siRNA blocked H2O2-induced reduction of alkaline phosphatase (ALP) activity and mineralization in human periodontal ligament fibroblasts(HPLFs), accompanied by restoration of Runx2, osteocalcin mRNA expression. These results suggest that activation of the Wnt/β-catenin pathway promotes the proliferation and mineralization of H_2_O_2_-exposed HPLFs by down-regulating Nrf2 (114). The expression of Runx2 in Nrf2 KO osteoblasts increased in a time-dependent manner at the mRNA and protein levels, and enhanced mineralization was detected (104). **Figure 6** briefly shows the effect of Nrf2 on osteoblasts.

## 4 Drugs related to the Nrf2 pathway

With the further clarification of the molecular mechanism for the regulation of Nrf2/ARE signaling pathway and its pathophysiological significance, drug development based on this pathway has attracted more and more attention. Plants are a rich source of compounds that activate Nrf2 transcription factors. Several natural compounds have been identified as electrophilic Nrf2 inducers, including sulforaphane, curcumin, resveratrol, and quercetin (115). Curcumin, the active ingredient in turmeric, is also found in small amounts in ginger; sulforaphane (SFN) is an isothiocyanate found in cruciferous vegetables; resveratrol has been found in dietary sources such as grapes, blueberries, peanuts and red wine. There are other plant compounds such as epigallocatechin gallate, ginseng fruit extract and green tea extract (116, 117). Studies have shown that all these drugs are not only good antioxidants, but also have a strong anti-inflammatory effect *via* Nrf2 induction in *in vivo* and/or *in vitro* experiments in periodontitis models. For example, baicalein, the main component of Scutellaria baicalensis, can regulate the Keap1-Nrf2 system through Keap1-independent and Keap1-dependent pathways (118). However, most of these compounds have not moved beyond proof-of-concept experiments, and it will take a long time to characterize their pharmacodynamic properties, clinical safety, and efficacy in non-communicable diseases. For the above-mentioned antioxidants, a variety of drug delivery methods have been developed to improve drug bioavailability and pharmacokinetics, such as solid dispersion, self-microemulsifying systems and nano-formulation. Commonly used nanoparticles are polylactic acid-glycolic acid copolymer (PLGA), chitosan and its derivatives solid lipid nanoparticles (SLN). For example, incorporation of curcumin into PLGA-based polymer nanoparticles resulted in a 640-fold increase in *in vitro* solubility; resveratrol-containing trimethylchitosan nanoparticles showed increased resveratrol uptake by Caco-2 cells in an *in vitro* cell monolayer model; SLN mediates lymphatic absorption and helps evade hepatic metabolism (119).

Extracellular vesicles can control inflammation by participating in immunomodulatory regulation of intercellular signaling, repair periodontal tissue and promote regeneration of periodontal tissue. Exos have the advantage over stem cell therapies in that they are relatively easy to preserve and sterilize, and can be stored for a long time without losing their properties (120). Studies have demonstrated that Exos is a natural nanoparticle delivery method for the treatment of a variety of infectious and immunological/inflammatory diseases. For example, exosome-treated rats repaired defects more efficiently by regenerating periodontal tissue, including newly formed bone and periodontal ligament (121); exosomes derived from gingival mesenchymal stem cells can target Wnt5a to regulate the RANKL/OPG system and inhibit periodontal bone loss (122). Nrf2 protects cells from the adverse effects of oxidation by activating antioxidant signaling pathways. Therefore, the application of Exosomal-Nrf2 as an emerging therapeutic tool is a promising approach.

## 5 Conclusion

The pathogenesis of periodontal disease has not been fully elucidated, but oxidative damage has been considered as an important pathogenesis. The Keap1-Nrf2 signaling pathway is the most important cellular antioxidant system, controlling the expression of various antioxidant enzymes. In this paper, the anti-oxidative, anti-apoptotic and anti-inflammatory effects of Nrf2 signaling pathway in periodontal disease were explored from a molecular perspective combined with experimental studies in recent years. Nrf2 has the potential to be a therapeutic target for periodontal disease. In recent years, a large number of experimental studies have shown that Nrf2 signaling pathway plays a beneficial role in alleviating periodontitis tissue damage, and some drugs targeting Nrf2 activation have made good progress in cell and animal experiments. However, the exact link between Nrf2 and oxidative stress and the exact mechanism by which it contributes to the treatment of periodontal disease are still unknown, and there is currently insufficient clinical evidence to confirm that Nrf2 activators play a practical and effective clinical role. Although many studies have achieved unprecedented results in elucidating the Keap1-Nrf2 signaling pathway, many key issues remain unresolved. For example: how many genes does the Nrf2 transcription factor control? When is activation and inhibition of Nrf2 appropriate? In addition, more studies are needed to assess dietary effects on the Keap1/Nrf2/ARE signaling pathway and the relationship between its physiological effects, as well as how to improve its bioavailability. Generally speaking, Nrf2 pathway is an important antioxidant stress pathway found *in vivo* at present. On the basis of existing research, a thorough analysis of the mechanism of Nrf2 signal network maintaining redox homeostasis, combined with early periodontal disease diagnosis, is helpful to find new methods to prevent and treat the periodontal disease, and ultimately improve the quality of life of patients with periodontal disease.

## Author contributions

FM, CL and ZL: Study conception and design; FM, CL, XJ, KC and JD: Drafting of manuscript; SL, SM and ZL: Critical revision. All authors contributed to the article and approved the submitted version.

## Funding

This work was funding by Science and Technology Projects in Guangzhou (grant No. 202102020020, 202201020536, 202201020069) and Fundamental Research for the Central Universities (grant No.21621410).

## Conflict of interest

The authors declare that the research was conducted in the absence of any commercial or financial relationships that could be construed as a potential conflict of interest.

## Publisher’s note

All claims expressed in this article are solely those of the authors and do not necessarily represent those of their affiliated organizations, or those of the publisher, the editors and the reviewers. Any product that may be evaluated in this article, or claim that may be made by its manufacturer, is not guaranteed or endorsed by the publisher.
